# Plant-Derived Polynucleotides/Polydeoxyribonucleotides in Skin Biomaterials: Delivery Platforms and Bioactivity Attribution

**DOI:** 10.3390/jfb17070351

**Published:** 2026-07-18

**Authors:** Hyun Joo Kim, Jin Woo Lee, Sanghyo Kim, Kuk Hui Son

**Affiliations:** 1 Research Institute, Sphebio Co., Ltd., 501-ho, 3, Achasan-ro 11ga-gil, Seongdong-gu, Seoul 04796, Republic of Korea; 2 Department of Health Sciences and Technology, GAIHST, Gachon University, 155, Gaetbeol-ro, Yeonsu-gu, Incheon 21999, Republic of Korea; jwlee@gachon.ac.kr; 3 Department of Molecular Medicine, College of Medicine, Gachon University, Incheon 21999, Republic of Korea; 4 Department of Bionanotechnology, Gachon University, 1342 Seongnamdaero, Sujeong-gu, Seongnam 13120, Republic of Korea; 5 Department of Thoracic and Cardiovascular Surgery, Gachon University Gil Medical Center, College of Medicine, Gachon University, Incheon 21565, Republic of Korea

**Keywords:** plant-derived polynucleotide, polydeoxyribonucleotide, PDRN, PN, DNA biopolymer, hydrogel, hyaluronic acid, chitosan, alginate, skin biomaterials, bioactivity attribution

## Abstract

In the fields of dermatology and skin biomaterials, polynucleotides (PN) and polydeoxyribonucleotides (PDRN) typically refer to deoxyribonucleic acid (DNA)-based polymers or heterogeneous DNA-fragment mixtures rather than ribonucleic acid (RNA) polynucleotides. Recently, plant-derived PN/PDRN preparations, sourced from callus, adventitious roots, and plant cell culture systems, have emerged as a promising animal-free material class. However, current evidence remains predominantly preclinical and should not be construed as demonstrating clinical equivalence to conventional animal-derived PDRN. This review synthesizes recent reports on plant-derived and other non-animal PN/PDRN and integrates them with delivery platform evidence relevant to skin biomaterials. We distinguish directly demonstrated findings from plant-derived preparations from inferences drawn from animal-derived PDRN, synthetic sequence-defined nucleic acids, or broader biomaterial delivery literature. The review further emphasizes operational molecular definitions, polymer length and fragment distribution reporting, DNA purity and integrity, RNA carryover, residual nucleoproteins and plant-derived macromolecules, free nucleotide/nucleoside or degradation product fractions, enzymatic degradation, delivery matrices, and hypothesis-matched controls. Available data suggest overlapping biological signatures with classical PDRN, including keratinocyte repair, fibroblast extracellular matrix (ECM) remodeling, and A2A receptor-associated readouts; however, A2A receptor dependency has not been directly established for most plant-derived PN/PDRN preparations and should be interpreted as a working mechanistic hypothesis unless perturbation experiments demonstrate pathway dependence. Direct head-to-head studies using matched DNA dose, molecular weight distribution, purity, delivery platform, and exposure conditions remain limited. Accordingly, plant-derived PN/PDRN should be evaluated as a source–process–structure–platform–function system rather than as a DNA fraction alone.

## 1. Introduction

In broad biochemical terminology, a polynucleotide (PN) can be composed of ribonucleotides or deoxyribonucleotides. In this review, however, PN/PDRN is used in the narrower dermatology and skin biomaterial sense and refers only to DNA-derived deoxyribonucleotide preparations unless explicitly stated otherwise [1,2]. PDRN denotes heterogeneous low- to medium-molecular-weight DNA fragment mixtures, whereas PN denotes longer DNA-derived polynucleotide fractions that may also contribute to viscoelastic, hydration, depot, or matrix-like behavior. Reported PN/PDRN preparations are commonly described across broad molecular size ranges, such as approximately 50–1500 kDa or approximately 50–2000 bp in parts of the PDRN literature, but these values should be treated as operational descriptors rather than universal chemical definitions [1,2]. Therefore, polymer length, molecular weight distribution, base pair range, strand state, purity, and degradation profile should be reported empirically for each preparation. The current PN/PDRN skin biomaterial literature also does not establish sequence-dependent effects in the way sequence-defined oligonucleotide drugs do. Most commercial or experimental PN/PDRN materials are heterogeneous DNA fractions; thus, their reported activity should be interpreted primarily through fragment size distribution, purity, degradation into nucleotides or nucleosides, receptor- or salvage-related signaling, residual contaminants, and delivery exposure rather than through an assumed nucleotide sequence [1,2].

Recent reviews have addressed PN/PDRN nomenclature [1,2], pharmacological mechanisms and tissue regeneration applications [3,4,5,6,7,8,9], and aesthetic outcomes or clinical practice patterns [10,11]. However, a focused analysis that systematically evaluates plant-derived PN/PDRN, source process variables, and delivery matrices remains scarce. In particular, there is a paucity of critical evaluation regarding the experimental evidence needed to deconvolve DNA-specific, source-specific, and platform-mediated hypotheses.

Underdeveloped areas include plant-derived PN/PDRN and their delivery. Plant calli, adventitious roots, and plant cell culture systems can generate animal-free DNA fractions [12,13,14,15,16,17,18,19,20]. However, plant source DNA preparations may differ from salmonid PDRN in molecular weight distribution, fragment length, strand state, residual protein or histone-like nucleoprotein content, RNA carryover, free nucleotide/nucleoside fraction, plant polysaccharides, phenolics, pigments, endotoxin-like contaminants, and batch-to-batch consistency [12,13,14,15,16,17,18,19,20,21,22]. Direct delivery platform studies using plant-derived PN/PDRN remain scarce. Therefore, HA matrices, alginate and oxidized alginate hydrogels, chitosan complexes, cellulose-based wound contact materials, topical hydrogels, and microneedle or post-procedure routes should be interpreted as candidate or benchmark delivery formats unless the plant-derived PN/PDRN itself is loaded, released, and tested under controlled conditions [23,24,25,26,27,28,29,30,31,32,33,34,35,36,37,38,39,40,41,42,43,44,45,46,47,48,49,50,51,52,53,54,55,56,57,58,59,60,61,62,63,64,65,66,67,68].

In this context, the biological response is generated by a molecule–platform–route system rather than by the DNA fraction alone; consequently, the same nominal DNA dose can yield different biological readouts when release kinetics, matrix binding, nuclease exposure, skin barrier bypass, and local retention differ [23,24,25,26,27,28,29,30,31,32,33,34,35,36,37,38,39,40,41,42,43,44,45,46,47,48,49,50,51,52,53,54,55,56,57,58,59,60,61,62,63,64,65,66,67,68].

The review therefore focused on plant-derived and other non-animal PN/PDRN evidence, delivery platforms relevant to skin biomaterials, and experimental designs that can separate DNA-specific activity from source-, carrier-, and route-mediated effects. Animal-derived PDRN is discussed as a benchmark for mechanism and comparator selection [3,4,5,6,7,8] and as a reference point for delivery design when conventional PN/PDRN platform studies are relevant [23,33,34,49,50,55].

## 2. Scope and Literature Search Strategy

A structured narrative approach was employed for this review. A comprehensive literature search was conducted across multiple databases, including PubMed/MEDLINE, Web of Science, Scopus, Google Scholar, and Crossref/DOI records, alongside selected official guidance and standard-setting websites, spanning the period from 1 January 1990, to 1 June 2026. The search strategy utilized key descriptors such as PN, PDRN, polydeoxyribonucleotide, polynucleotide, plant-derived PDRN, callus-derived PDRN, plant PN, plant cell culture, skin regeneration, wound healing, skin barrier, hydrogel, hyaluronic acid, alginate, chitosan, nanocellulose, microneedle, and cosmetic science. While the 1990 baseline effectively captures the inception and evolution of modern PN/PDRN pharmacology, skin regeneration, and biomaterials research, this synthesis is deliberately weighted toward recent advancements in plant-derived or non-animal PN/PDRN variants and their corresponding delivery platforms. Consequently, older literature within this chronological scope was referenced selectively, primarily to establish foundational context regarding PN/PDRN terminology, hyaluronan or alginate matrices, adenosine biology, wound-healing mechanisms, and nucleic acid sensing.

**Figure 1:**
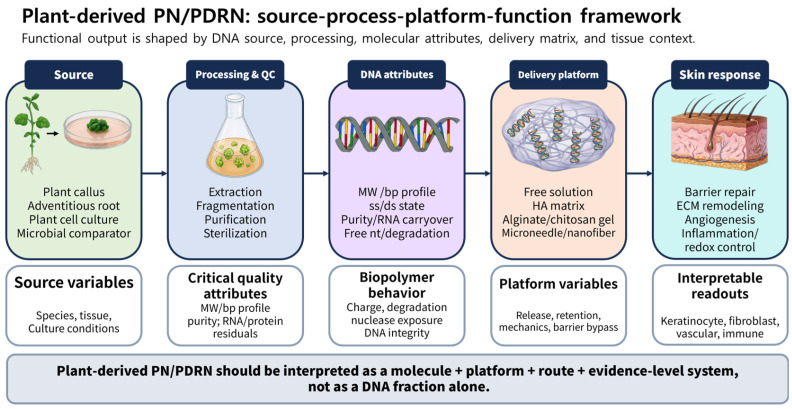
Source–process–platform–function framework for plant-derived PN/PDRN. Source and process variables are supported by plant source, microbial source, and plant cell culture literature [12,13,14,15,16,17,18,19,20,21,22,69], whereas platform variables are supported by PN/PDRN delivery and biomaterial studies [23,24,25,26,27,28,29,30,31,32,33,34,35,36,37,38,39,40,41,42,43,44,45,46,47,48,49,50,51,52,53,54,55,56,57,58,59,60,61,62,63,64,65,66,67,68].

The included sources were rigorously classified into four distinct evidence categories: (i) direct studies on plant-derived PN/PDRN focusing on skin cells or skin regeneration; (ii) investigations of other non-animal DNA fragments serving as non-salmon comparators; (iii) studies on animal-derived PDRN/PN utilized strictly as mechanistic or clinical benchmarks; and (iv) broader literature on nucleic acid delivery or skin biomaterials deployed to interpret platform-mediated effects. Conversely, publications were excluded if they focused on non-skin applications—such as ophthalmology, dentistry, musculoskeletal, or systemic systems—unless they offered fundamental insights into PDRN mechanisms or delivery strategies. Furthermore, non-peer-reviewed promotional materials and studies lacking a sufficient characterization of the tested material were excluded. Due to the heterogeneous nature and predominantly preclinical status of the available evidence on plant-derived PN/PDRN, a formal meta-analysis was not attempted (Figure 1).

## 3. Molecular Definition and Source-Process Variables

For this review, PN/PDRN terminology is used operationally rather than as a fixed chemical taxonomy. PDRN is used for purified or DNA-enriched low- to medium-molecular-weight deoxyribonucleotide fragment mixtures, whereas PN is used for longer DNA-derived polynucleotide fractions that may additionally contribute to rheological, hydration, depot, or matrix-like behavior [1,2,9]. Because source material and fragmentation processes can shift the molecular size profile, the term PN or PDRN alone does not define polymer length, bioactivity, or delivery behavior. Molecular weight distribution, base-pair range, and strand state should therefore be treated as core descriptors rather than optional quality control details.

For plant-derived DNA fractions, nomenclature is inseparable from analytical characterization [1,2,12,13,14,15,16,17,18,19,20,21,22]. A plant source preparation should be regarded as a DNA-enriched heterogeneous fraction unless batch-level composition confirms DNA fragment identity and quantifies or excludes residual RNA, DNA-bound or co-purified proteins, free nucleotides and nucleosides, short oligonucleotides, degradation products, plant polysaccharides, phenolics, pigments, salts, endotoxin/pyrogen burden, and bioburden [21,22,69,70]. These attributes are biologically interpretive variables rather than only manufacturing descriptors: DNA mass defines nominal dose; molecular weight and base-pair distributions influence diffusion, nuclease susceptibility, release kinetics, and tissue retention; strand state can alter matrix binding and degradation; and free nucleotides or nucleosides can mimic downstream PDRN degradation and contribute to adenosine-related or salvage pathway effects independently of intact DNA fragments [12,13,14,15,16,17,18,19,20,21,22,69,70].

Synthetic sequence-defined nucleic acids should be distinguished from heterogeneous PN/PDRN preparations. Synthetic CpG oligodeoxynucleotides and synthetic double-stranded RNA mimetics such as polyinosinic:polycytidylic acid [poly (I:C)] can activate sequence- or structure-specific innate immune receptors, including Toll-like receptor 9 (TLR9) or Toll-like receptor 3 (TLR3), and therefore represent a different mechanistic class from heterogeneous DNA-fragment PN/PDRN preparations used in skin biomaterials [71,72]. Within the literature scope of this review, no robust skin biomaterial study was identified showing that synthetic PDRN-like mixtures reproduce the same biological profile as plant-derived or salmon-derived PDRN under matched molecular weight, purity, and delivery conditions. Sequence-specific PN/PDRN activity should therefore be proposed only when defined sequences, motif analysis, or sequence-matched and scrambled controls are experimentally tested.

The purity of PN/PDRN preparations cannot be inferred from nomenclature alone. Histones and other DNA-bound proteins would be expected to be largely removed by effective deproteinization and purification, but their residual presence has not been systematically examined in most plant-derived PN/PDRN studies. Similarly, residual RNA, free nucleotides, deoxyribonucleotides, nucleosides, short oligonucleotides, and degradation products may be present depending on source tissue, extraction, fragmentation, purification, sterilization, storage, and nuclease exposure [21,22,68,69,70]. This distinction is important because residual nucleoproteins or RNA may activate non-DNA mechanisms, while free nucleotides/nucleosides or pre-existing degradation products may contribute to A2A-related or salvage metabolism readouts without requiring intact PN/PDRN activity [3,4,5,6,7,8,21,22,69,70,73,74,75,76,77,78]. DNA-only or intact-fragment mechanisms should therefore be proposed only when supported by RNase/DNase digestion controls, fluorometric DNA/RNA quantification, electrophoretic or chromatographic fragment profiling, BCA/Bradford protein assays, carbohydrate and phenolic assays, HPLC/LC-MS profiling of nucleotides/nucleosides, and endotoxin or sterility testing [21,22,68,69,70].

Delivery status is part of this operational definition. A free DNA fraction in cell culture is not functionally equivalent to the same fraction embedded in HA, alginate, or chitosan systems [23,33,34,49,50,55], adsorbed onto nanocellulose-based wound-contact materials [56,57,58,59], or delivered through microneedle-based routes [60,61,62,63,64,65,66,67]. Hydrogel and nucleic acid delivery principles indicate that these platforms can change release, retention, degradation, and local exposure even when nominal DNA doses are identical [23,24,25,26,27,28,29,30,31,32,33,34,35,36,37,38,39,40,41,42,43,44,45,46,47,48,49,50,51,52,53,54,55,56,57,58,59,60,61,62,63,64,65,66,67,68]. Operational definitions and reporting elements were organized in Table 1.

**Table 1:** Operational definitions and reporting elements for studies on plant-derived PN/PDRN.

Term	Operational Meaning in This Review	Why It Matters for Delivery	Minimum Reporting Elements
PDRN	DNA-derived heterogeneous low- to medium-molecular-weight deoxyribonucleotide fragment mixture; commonly described in broad PDRN literature ranges such as ~50–1500 kDa or ~50–2000 bp, but not defined by a universal cutoff [1,2].	May diffuse rapidly, degrade into nucleotides/nucleosides, require local retention or nuclease protection, and show bioactivity that depends on fragment-size distribution rather than name alone [1,2,3,4,5,6,7,8,70].	DNA mass, MW distribution, bp range, strand state, nuclease stability, purity ratios, RNA carryover, free nucleotide/nucleoside fraction, and degradation profile [1,2,21,22,70].
PN	Longer DNA-derived polynucleotide fraction or high-MW preparation used in dermatology/skin biomaterials; not used here for RNA polymers unless explicitly specified [1,2,9,10,11].	May contribute to viscoelasticity, hydration, depot behavior, matrix interaction, or tissue residence in addition to biochemical readouts [1,2,9,10,11].	MW threshold or distribution, rheology, viscosity, degradation, tissue residence, dose as DNA mass, and formulation context [1,2,9,10,11].
Plant-derived PN/PDRN	DNA-enriched fraction extracted from callus, adventitious root, plant cell culture, or plant tissue [12,13,14,15,16,17,18,19,20,21,22].	Plant residuals, RNA carryover, residual nucleoproteins, phenolics, polysaccharides, and degradation products can confound antioxidant, inflammatory, and wound repair outcomes [12,13,14,15,16,17,18,19,20,21,22,69].	Species, tissue/cell source, culture conditions, extraction, fragmentation, purification, sterilization, residual protein/nucleoprotein, RNA, polysaccharide, phenolic content, endotoxin/pyrogen, and sterility [12,13,14,15,16,17,18,19,20,21,22,69,70].
Delivery-controlled PN/PDRN	DNA fraction combined with a defined carrier, matrix, scaffold, or device [23,24,25,26,27,28,29,30,31,32,33,34,35,36,37,38,39,40,41,42,43,44,45,46,47,48,49,50,51,52,53,54,55,56,57,58,59,60,61,62,63,64,65,66,67,68].	Observed effects may reflect DNA, carrier, route, release profile, nuclease protection, or matrix–cell interactions [23,24,25,26,27,28,29,30,31,32,33,34,35,36,37,38,39,40,41,42,43,44,45,46,47,48,49,50,51,52,53,54,55,56,57,58,59,60,61,62,63,64,65,66,67,68].	Platform composition, release kinetics, released-fraction DNA integrity, carrier-only control, free-DNA control, loaded-platform arm, local retention, and route of administration [23,24,25,26,27,28,29,30,31,32,33,34,35,36,37,38,39,40,41,42,43,44,45,46,47,48,49,50,51,52,53,54,55,56,57,58,59,60,61,62,63,64,65,66,67,68].

## 4. Plant-Derived and Other Non-Animal PN/PDRN Evidence

Currently, the strongest skin-related evidence for plant-derived PN/PDRN comes from *Panax ginseng* adventitious root PDRN, *Hibiscus sabdariffa* callus PDRN, *Gynostemma pentaphyllum* callus PDRN, *Paeonia lactiflora* callus-derived PN, and *Saussurea involucrata*-derived PDRN [12,13,14,15,16]. These reports are preclinical, largely based on skin cells, three-dimensional (3D) skin models, transcriptomic analyses, or early mechanistic assays. A microbially derived PDRN preparation from *Lactobacillus rhamnosus* provides a useful non-animal comparator, although it is not plant-derived [69].

*Panax ginseng* adventitious root PDRN was associated with increased keratinocyte and fibroblast proliferation, supported re-epithelialization in a 3D skin model, increased fibronectin, filaggrin, Ki-67, B-cell lymphoma 2 (Bcl-2), inhibin βA, and cyclin D1, and was linked to A2A receptor-like activity and focal adhesion kinase (FAK)/protein kinase B (AKT)/mitogen-activated protein kinase (MAPK) phosphorylation [12]. *Hibiscus sabdariffa* callus PDRN improved human keratinocyte repair and upregulated nuclear factor erythroid 2-related factor 2 (Nrf2)-related antioxidant and repair markers, including superoxide dismutase 2 (SOD2), catalase (CAT), aquaporin 3 (AQP3), keratinocyte growth factor (KGF), vascular endothelial growth factor (VEGF), and matrix metalloproteinase 9 (MMP9) [13]. *Gynostemma pentaphyllum* callus PDRN increased keratinocyte viability, demonstrated antioxidant and wound-healing effects, and modulated barrier markers, including filaggrin (FLG), involucrin (IVL), and claudin-1 (CLDN1) [14]. The most detailed dermal fibroblast mechanism among the current plant-source reports was obtained with *Paeonia lactiflora* callus-derived PN. It increased cell viability and pro-collagen Iα1 secretion and was associated with A2A receptor expression, cyclic adenosine monophosphate (cAMP)/protein kinase A (PKA)/cAMP response element-binding protein (CREB), Cyclin D1/retinoblastoma protein (Rb)/proliferating cell nuclear antigen (PCNA), nuclear factor κB (NF-κB)/matrix metalloproteinase (MMP) suppression, transforming growth factor β (TGF-β)/Smad signaling, and collagen/elastin deposition [15]. *Saussurea involucrata* PDRN further supports the feasibility of plant-source DNA fractions, but available mechanistic and delivery data remain limited [16].

Collectively, current plant-derived and other non-animal PN/PDRN studies support feasibility but not clinical equivalence [12,13,14,15,16,69] (Table 2). Direct clinical studies of plant-derived PN/PDRN for skin indications were not identified. The current evidence should therefore be interpreted as hypothesis-generating rather than confirmatory. The main strengths of the available studies are their use of human skin-relevant cells, barrier- or ECM-related markers, several repair-associated readouts, and, in some cases, 3D skin or pathway-associated analyses. The main limitations are incomplete molecular characterization of the tested DNA fractions, inconsistent reporting of fragment distribution and degradation products, limited residual-contaminant profiling, scarce process blanks or DNase/RNase-treated controls, lack of matched animal-derived PDRN comparators, limited delivery platform testing, and little independent replication across laboratories or production lots [12,13,14,15,16,17,18,19,20,21,22,69,70] (Table 3).

These limitations affect bioactivity attribution. Increases in proliferation, migration, antioxidant markers, collagen-related proteins, or barrier markers should be read as preliminary biological signals unless DNA specificity, source specificity, and platform contribution are separated experimentally [12,13,14,15,16,17,18,19,20,21,22,69,70]. A rigorous study-quality framework should therefore consider material characterization, comparator adequacy, control architecture, delivery platform separation, model relevance, mechanism perturbation, effect-size reporting, and repeat lot reproducibility before assigning a generalizable PN/PDRN mechanism or translational interpretation [12,13,14,15,16,17,18,19,20,21,22,69,70].

**Table 2:** Study-level critical appraisal of current plant-derived and other non-animal PN/PDRN skin studies.

Study/Source	Main Strength	Main Study-Quality Limitation	Reproducibility Gap	Interpretation Level
*Panax ginseng* adventitious root PDRN [12]	Includes keratinocyte/fibroblast readouts, 3D skin-related repair markers, and A2A-like plus FAK/AKT/MAPK-associated signals.	Limited matched animal-derived PDRN comparison; incomplete separation of DNA-specific activity from source/process effects and delivery conditions.	Independent laboratory replication and repeat-lot testing remain limited.	Promising preclinical signal; mechanism remains a working hypothesis.
*Hibiscus sabdariffa* callus PDRN [13]	Provides keratinocyte repair and Nrf2-centered antioxidant/repair marker data.	Redox and antioxidant readouts may be confounded by residual phenolics or other callus-derived components unless process blanks and contaminant profiling are included.	Repeat-batch and independent replication are needed.	Suggestive skin-repair signal; DNA-specific attribution remains incomplete.
*Gynostemma pentaphyllum* callus PDRN [14]	Links plant-derived PDRN to keratinocyte viability and barrier-associated markers such as FLG, IVL, and CLDN1.	Tissue-level barrier function is not fully established; TEER/permeability and inflammatory-damage models are needed.	Lot-to-lot reproducibility and source-matched controls remain limited.	Barrier-relevant preclinical signal; translational strength remains limited.
*Paeonia lactiflora* callus-derived PN [15]	Provides the most detailed dermal fibroblast mechanism among current plant-source reports, including ECM and pathway-associated markers.	Limited in vivo or ex vivo validation; no same-platform animal-derived PDRN comparator; pathway dependency requires stronger perturbation controls.	Independent replication and repeated production lots are needed.	Strongest mechanistic plant-source report, but not yet translationally confirmatory.
*Saussurea involucrata* PDRN [16]	Supports feasibility of plant-source DNA fractions for skin-cell activity.	Mechanistic depth, molecular characterization, delivery evidence, and comparator controls remain limited.	Batch-level and independent-laboratory reproducibility are untested.	Early feasibility evidence.
*Lactobacillus rhamnosus* PDRN [69]	Useful non-animal comparator with small-fragment and A2A-associated FAK/AKT/MAPK signaling data.	Not plant-derived; source biology and residual composition differ from plant preparations.	Needs matched comparison with plant and animal PDRN under equivalent DNA size and dose.	Comparator evidence, not direct plant-derived evidence.

**Table 3:** Representative plant-derived and other non-animal PN/PDRN studies relevant to skin biology.

Source	Model	Main Reported Effects	Delivery Gap
*Panax ginseng* adventitious root PDRN [12]	HaCaT/keratinocytes, fibroblasts, 3D artificial skin	Proliferation, re-epithelialization, FLG/FN/Ki-67/Bcl-2/cyclin D1, A2A-like and FAK/AKT/MAPK signaling	Direct hydrogel/matrix delivery data remain limited.
*Hibiscus sabdariffa* callus PDRN [13]	Human keratinocytes	Nrf2-centered antioxidant and repair axis; AQP3, KGF, VEGF, SOD2, CAT, MMP9	Residual antioxidant phytochemical effects require controls.
*Gynostemma pentaphyllum* callus PDRN [14]	Keratinocyte viability and transcriptomic/barrier analysis	Antioxidant, anti-inflammatory, wound-healing and barrier-marker effects; FLG, IVL, CLDN1	Needs matched source and platform controls.
*Paeonia lactiflora* callus PN [15]	Human dermal fibroblasts	A2A receptor-associated expression, cAMP/PKA/CREB, cell cycle progression, NF-κB/MMP reduction, TGF-β/Smad, collagen/elastin deposition	Strong mechanism; platform and in vivo delivery evidence needed.
*Saussurea involucrata* PDRN [16]	Human skin-cell assays	Proliferation, migration, collagen synthesis, MMP1 suppression	QC and mechanistic depth need expansion.
*Lactobacillus rhamnosus* PDRN [69]	Non-animal microbial comparator	Small fragments; A2A receptor-associated FAK/AKT and MAPK signaling	Useful comparator but not plant-derived.

### Mechanistic Relationship Between Plant-Derived PN/PDRN and Animal-Derived PDRN

The mechanistic relationship between plant-derived PN/PDRN and classical animal-derived PDRN remains unresolved. Animal-derived PDRN is commonly interpreted through degradation into nucleotides/nucleosides, adenosine A2A receptor-related signaling, and nucleotide salvage [1,2,3,4,5,6,7,8,73,74,75,76,77,78]. This framework is useful for comparator selection but should not be treated as direct proof that plant-derived PN/PDRN operates through the same pathway, because matched comparisons between plant-derived PN/PDRN and animal-derived PDRN remain scarce [12,13,14,15,16,69].

In this review, the term “A2A receptor-associated” is used deliberately to distinguish marker-level or pathway-associated observations from demonstrated receptor dependency. A mechanism is considered demonstrated only when functional perturbation experiments, such as selective A2A receptor antagonism, receptor knockdown or rescue, DNA-degradation controls, and orthogonal downstream readouts, show that the biological effect depends on that pathway [3,4,5,6,7,8,12,15,69,73,74,75,76,77,78]. By contrast, changes in A2A receptor expression, A2A-like activity, cAMP/PKA/CREB signaling, or FAK/AKT/MAPK phosphorylation are interpreted as pathway-associated signatures unless causality is experimentally tested.

Several plant-derived PN/PDRN studies have reproduced aspects of these mechanistic signatures. Among them, *Panax ginseng* adventitious root PDRN provides the closest current example of animal PDRN-like activity because it has been associated with A2A receptor-like activity, FAK/AKT/MAPK phosphorylation, keratinocyte and fibroblast proliferation, re-epithelialization, and increases in filaggrin, fibronectin, Ki-67, Bcl-2, and cyclin D1 levels [7,12]. *Paeonia lactiflora* callus-derived PN provides the most detailed dermal fibroblast mechanism, linking plant PN to A2A receptor expression, cAMP/PKA/CREB activation, Cyclin D1/Rb/PCNA cell-cycle signaling, NF-kB/MMP suppression, TGF-β/Smad activation, and collagen/elastin accumulation [15].

Other plant-source reports suggest potentially complementary, source-sensitive mechanisms, rather than the simple replication of animal-derived PDRN biology. *Hibiscus sabdariffa* callus PDRN promotes an antioxidant repair axis involving Nrf2, SOD2, CAT, AQP3, KGF, VEGF, and MMP9 in keratinocytes [13]. *Gynostemma pentaphyllum* callus PDRN enhances keratinocyte viability, antioxidant and wound-healing activities, and barrier-related changes in FLG, IVL, and CLDN1 [14]. *Saussurea involucrata* PDRN supports broader plant-source feasibility through proliferation, migration, collagen synthesis, and MMP1 suppression; however, its mechanistic depth remains limited [16].

Therefore, the available evidence does not support either a completely distinct mechanism for plant-derived PN/PDRN or full mechanistic equivalence with animal-derived PDRN. More conservatively, current data show partially overlapping biological and pathway-associated signatures, including MAPK/AKT activation, cell proliferation, ECM remodeling, barrier repair, and A2A receptor-associated readouts in selected studies. Additional plant-source signals, such as Nrf2/redox regulation, AQP3-mediated hydration, and barrier-gene modulation, may reflect DNA fragment activity, plant source-specific DNA features, residual phytochemicals or polysaccharides, or formulation context [12,13,14,15,16].

Direct, head-to-head evidence remains sparse. Most plant-derived PN/PDRN studies do not match animal-derived PDRN in DNA mass, molecular-weight distribution, fragment length, purity, delivery platform, or exposure time [12,13,14,15,16,23,33,34,49,50,55,69]. A2A receptor involvement is supported indirectly or partially in several reports, but full pathway dependence has not been established [1,7,12,15,69]. Therefore, A2A receptor-related findings should be reported as working mechanistic hypotheses unless receptor antagonism or knockdown, DNA-degradation controls, nucleotide/nucleoside profiling, and orthogonal pathway markers demonstrate dependency under matched molecular and delivery conditions [3,4,5,6,7,8,73,74,75,76,77,78]. The evidence grading and requirements based on the proposed mechanism in the PN/PDRN study were as shown in Table 4.

**Table 4:** Evidence grading of proposed mechanisms in animal-derived, plant-derived, and other non-animal PN/PDRN studies.

Proposed Mechanism or Readout	Evidence in Animal-Derived PDRN	Evidence in Plant-Derived/Other Non-Animal PN/PDRN	Current Interpretation	Experiments Required to Establish Causality
A2A receptor signaling and nucleotide salvage	Canonical framework in animal-derived PDRN literature for wound repair, inflammation control, angiogenesis, and fibroblast activation [1,2,3,4,5,6,7,8,73,74,75,76,77,78].	Panax and Paeonia report A2A-like or A2A-associated signatures; microbial PDRN also reports A2A-associated FAK/AKT/MAPK activity [7,12,15,69].	Working hypothesis for plant-derived PN/PDRN; not a demonstrated universal mechanism.	A2A antagonist or knockdown, rescue experiments, DNase-degraded controls, nucleotide/nucleoside profiling, matched animal PDRN comparator, and matched DNA dose/fragment size.
ECM remodeling and fibroblast response	Animal-derived PN/PDRN are associated with fibroblast proliferation, collagen synthesis, MMP regulation, and wrinkle-related ECM remodeling [1,2,3,4,5,6,7,8,9,79,80].	Paeonia PN reports pro-collagen Ialpha1, TGF-beta/Smad, collagen/elastin deposition, and NF-kB/MMP suppression [15].	Strong preclinical association, but not clinical or source-equivalence evidence.	Same-platform animal PDRN comparator, pathway perturbation, repeat lots, and ex vivo or 3D skin validation.
FAK/AKT/MAPK and cAMP/PKA/CREB signaling	Reported downstream pathways in conventional PDRN and adenosine-related literature [3,4,5,6,7,8,73,74,75,76,77,78].	Panax and Paeonia show pathway-associated phosphorylation or marker changes [12,15].	Pathway-associated signature; dependency remains incompletely proven.	Pathway inhibitors, time-course analysis, orthogonal downstream markers, repeat lots, and matched controls.
Nrf2/redox and inflammatory modulation	Less central than A2A/salvage in the classical PDRN framework; redox/inflammatory modulation is reported in broader PDRN literature [3,4,5,6,7,8,81,82].	Hibiscus and Gynostemma emphasize Nrf2, SOD2, CAT, antioxidant activity, and barrier/inflammatory markers [13,14].	Possible plant-source-associated biology or residual phytochemical effect; DNA-specificity remains uncertain.	Process blanks, phenolic quantification, Nrf2/NF-kB perturbation, DNase/RNase controls, and contaminant-matched controls.
Barrier repair and wound-repair markers	Animal PDRN literature supports re-epithelialization, wound closure, and epidermal repair benchmarks [3,4,5,6,7,8].	Panax, Gynostemma, and Hibiscus report FLG, IVL, CLDN1, AQP3, VEGF/KGF, or repair markers [12,13,14].	Barrier- and repair-relevant biological signal; tissue-level function requires validation.	3D skin, TEER/permeability, barrier-damage models, endothelial/macrophage assays, ex vivo wounds, and repeat-lot testing.

## 5. Delivery Platforms as Determinants of Plant PN/PDRN Function

Direct delivery platform studies using plant-derived PN/PDRN remain scarce [12,13,14,15,16,69]. Accordingly, alginate-PDRN, oxidized alginate-PDRN, chitosan-PDRN, and HA-PN studies are interpreted as animal-derived or conventional DNA-fragment delivery benchmarks rather than as direct proof for plant-derived systems [23,33,34,49,50,55].

**Figure 2:**
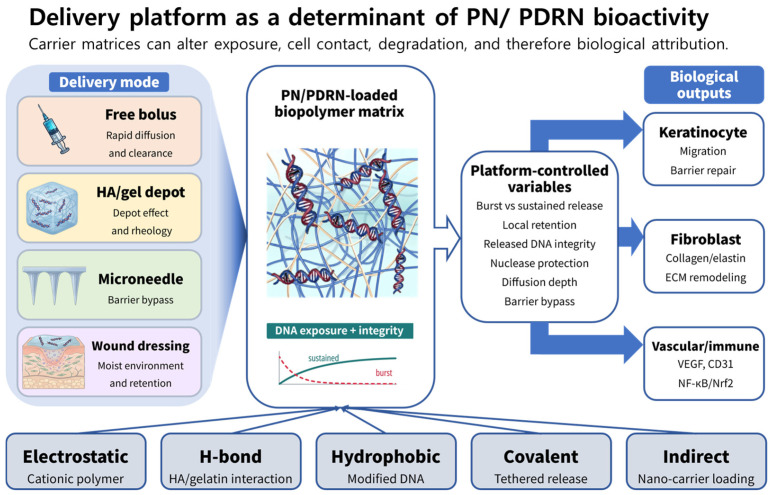
Delivery platform as a determinant of PN/PDRN bioactivity. Matrix binding, release kinetics, local retention, nuclease protection, diffusion depth, and barrier bypass can alter DNA exposure and biological readouts. For plant-derived PN/PDRN, most platform concepts remain benchmark or candidate-platform evidence unless plant-derived DNA is directly loaded and tested under controlled conditions [23,24,25,26,27,28,29,30,31,32,33,34,35,36,37,38,39,40,41,42,43,44,45,46,47,48,49,50,51,52,53,54,55,56,57,58,59,60,61,62,63,64,65,66,67,68].

Delivery constraints are central to PN/PDRN because DNA fragments are hydrophilic, negatively charged, and susceptible to enzymatic degradation [68,70]. Skin exposure adds an additional barrier because the stratum corneum restricts the penetration of large hydrophilic molecules and contains DNA-degrading activity; DNase 2 has been identified as a main DNA-degrading enzyme of the stratum corneum [60,61,62,63,64,65,66,67,70]. For plant-derived PN/PDRN, delivery platforms are therefore relevant not only for convenience of administration but also for release kinetics, nuclease protection, surface residence, and whether DNA remains intact long enough to reach keratinocytes or dermal fibroblasts (Figure 2).

Alginate-PDRN hydrogels provide one of the clearest examples of delivery-dependent PDRN activity. In a diabetic wound model, comparison of saline, free PDRN injection, alginate-only hydrogel, and alginate-PDRN hydrogel showed that both the matrix and sustained PDRN delivery contributed to wound repair, and that the loaded hydrogel outperformed free PDRN [33]. Oxidized alginate-PDRN systems extend this concept by adding ROS-scavenging, anti-inflammatory, anti-apoptotic, and angiogenesis-related functions, making attribution to the DNA fraction alone more difficult [34].

Chitosan-based PDRN delivery exploits the electrostatic interactions between cationic polysaccharide chains and negatively charged DNA. Chitosan-PDRN polyplexes and chitosan derivative-crosslinked hydrogels have been evaluated for wound healing and controllable release; however, chitosan can also influence hemostasis, antimicrobial activity, cell migration, and local inflammation [49,50,51,52,53,54].

HA-PN matrices are particularly relevant to cosmetic skin boosters because HA contributes to hydration, viscoelasticity, injectability, tissue spreading, and local depot behavior [23,24,25,26,27,28,29,30,31,32]. In studies on HA-PN fillers, improvements in fibroblast migration, collagen synthesis, and tissue durability should thus be interpreted as composite formulation outcomes, unless the PN-only- and HA-only arms are tested under matched dose and exposure conditions [23].

Nanocellulose and bacterial cellulose offer prospective routes toward fully or largely bio-based plant PN scaffolds because they provide high water content, conformability, and mechanical support for wound contact materials [56,57,58,59]. Microneedles and post-procedure application strategies are also relevant because the intact stratum corneum limits the penetration of large hydrophilic macromolecules; physical barrier bypass can determine whether DNA reaches the viable epidermis or dermis [60,61,62,63,64,65,66,67]. The role of plant PN/PDRN, interpretive risk, and key controls for each platform are summarized in Table 5.

**Table 5:** Delivery platform variables and control considerations for interpreting plant-derived PN/PDRN bioactivity.

Platform	Potential Role for Plant PN/PDRN	Main Interpretive Risk	Key Controls
Free solution	Defines baseline DNA exposure in cell culture or injection.	Rapid dilution/degradation; not representative of commercial matrices.	Vehicle and dose–response; DNA integrity [1,2,3,4,5,6,7,8,12,13,14,15,16,69].
HA matrix	Injectable or topical hydration matrix with rheology and depot behavior.	HA contributes biological and mechanical effects.	HA-only, PN-only, HA-PN, matched rheology/dose [23,24,25,26,27,28,29,30,31,32].
Alginate/oxidized alginate hydrogel	Wound dressing or depot with sustained release and moist wound environment.	Alginate or oxidized alginate can independently affect repair, ROS, and inflammation.	Free PN, alginate-only, loaded hydrogel, release profile [33,34,44,45,46,47,48].
Chitosan polyplex or hydrogel	Electrostatic DNA complexation, nuclease protection, wound retention.	Chitosan itself can alter hemostasis, bacteria, uptake, and immune signaling.	Chitosan-only, free PN, loaded chitosan, cytocompatibility [49,50,51,52,53,54].
Nanocellulose scaffold	Bio-based skin-contact scaffold for hydrated presentation of plant DNA.	Scaffold hydration/mechanics may dominate outcomes.	Scaffold-only, PN-only, loaded scaffold [56,57,58,59].
Microneedle/post-procedure delivery	Barrier bypass and localized epidermal/dermal deposition.	Needling injury itself may induce repair.	Blank microneedle, topical PN, PN-loaded microneedle [60,61,62,63,64,65,66,67].

## 6. Bioactivity Attribution: Hypothesis-Driven Experimental Design

**Figure 3:**
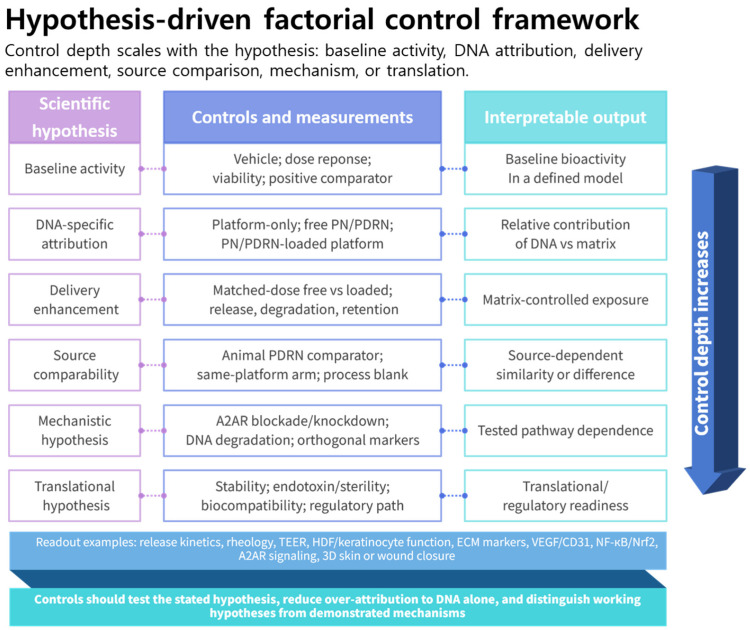
Hypothesis-driven factorial control framework for plant-derived PN/PDRN-related studies. Control depth scales with the tested hypothesis: baseline activity, DNA-specific attribution, delivery enhancement, source comparison, mechanism testing, or translational readiness [12,13,14,15,16,22,23,33,34,49,50,55,69,70,73,74,75,76,77,78,79,80,81,82,83,84,85,86,87,88,89,90] (Abbreviations in the figure: CQA, critical quality attribute; MW, molecular weight; QC, quality control; TEER, transepithelial electrical resistance).

The required experimental design depends on the biological hypothesis (Figure 3). A baseline activity hypothesis can be supported by vehicle controls, dose–response data, viability assays, and a positive comparator [12,13,14,15,16,69]. A DNA-specific hypothesis requires carrier-only and free-DNA arms. A delivery-enhancement hypothesis requires matched-dose comparisons between free PN/PDRN and PN/PDRN-loaded platforms together with release or retention data [23,33,34,49,50,55].

For plant-derived PN/PDRN, source comparability introduces another layer of complexity. If a study hypothesizes equivalence or superiority to animal-derived PDRN, the comparison should use a matched DNA dose, matched or reported fragment distribution, and, ideally, the same delivery platform [12,13,14,15,16,23,33,34,49,50,55,69]. Process blanks, degraded DNA controls, or contaminant-aware controls are important when residual plant proteins, polysaccharides, phenolic compounds, pigments, salts, or endotoxin-like materials can affect the same outcomes [12,13,14,15,16,17,18,19,20,21,22,69,70].

Mechanistic hypotheses should be directly tested rather than inferred from marker changes alone. For A2A receptor-related hypotheses, receptor expression or downstream phosphorylation should not be considered sufficient evidence of receptor-mediated activity. A minimum mechanistic design should include selective A2A receptor antagonism or receptor knockdown, matched intact and DNase-degraded PN/PDRN fractions, nucleotide/nucleoside profiling of degradation products, and orthogonal downstream readouts such as cAMP/PKA/CREB, FAK/AKT/MAPK, VEGF, inflammatory cytokines, and ECM markers [3,4,5,6,7,8,12,15,69,73,74,75,76,77,78]. Nrf2 activation, NF-kB suppression, TGF-beta/Smad modulation, VEGF-mediated angiogenesis, and ECM/barrier readouts should likewise be tested with pathway-specific markers or inhibitors appropriate to the source and cell model [13,14,15,79,80,81,82,83,84,85]. Without perturbation-based evidence, these findings should be described as pathway-associated signatures rather than demonstrated mechanisms. The core control factors based on the hypothesis in PN/PDRN biomaterial research are summarized in Table 6.

The alginate-PDRN hydrogel study is a useful model because saline, free PDRN, alginate-only, and alginate-PDRN arms enabled separation of baseline, DNA, carrier, and loaded-platform effects [33]. HA-PN and chitosan-PDRN systems are similarly informative when component controls are included; however, results from composite products should not be attributed to PN/PDRN alone unless such controls are present [23,49,50].

**Table 6:** Hypothesis-driven control considerations for plant-derived PN/PDRN biomaterial studies.

Hypothesis	Core Controls	Additional Elements When the Hypothesis Expands	Interpretation Enabled
Plant PN/PDRN has activity	Vehicle, dose response, viability, positive comparator	Purity profile, repeat batch	Baseline activity in a defined model [12,13,14,15,16,69]
Effect is DNA-specific	Vehicle, carrier-only, free PN/PDRN, loaded platform	DNase-treated DNA, process blank	Relative contribution of DNA vs. matrix [23,33,34,41,42,43,49,50,55,68]
Delivery improves performance	Matched-dose free PN/PDRN vs. loaded platform; carrier-only	Release, degradation, retention, nuclease challenge	Added value of matrix-controlled exposure [23,33,34,41,42,43,49,50,55,68]
Plant-derived PN/PDRN is comparable to animal-derived PDRN	Plant PN/PDRN vs. animal PDRN at matched dose	Same-platform comparison, fragment-size matching	Source-dependent similarity or difference [1,2,3,4,5,6,7,8,12,13,14,15,16,69]
Defined mechanism is hypothesized	Pathway markers and antagonist/inhibitor	Knockdown/rescue or orthogonal model	Mechanistic rather than descriptive attribution [3,4,5,6,7,8,12,13,14,15,16,69,73,74,75,76,77,78,81,82]
Skin-contact translational biomaterial is hypothesized	Cytotoxicity, irritation-relevant model, release/stability screening	Release/stability screening, sterility/endotoxin screening, and animal-study reporting elements when in vivo models are used	Early translation readiness [23,33,34,41,42,43,49,50,55,86,87,88,89,90]

## 7. Critical Quality Attributes and Reporting Elements

Studies on plant-derived PN/PDRN should connect molecular quality attributes with biological interpretation and delivery performance. Reporting DNA concentration alone is insufficient because quality attributes are not merely manufacturing descriptors. DNA mass determines nominal dose, whereas molecular-weight distribution, base-pair range, DNA integrity, strand state, RNA carryover, free nucleotide/nucleoside fraction, residual nucleoproteins, plant macromolecules, endotoxin/pyrogen burden, sterility, and degradation profile determine how the material diffuses, degrades, binds matrices, is released, and interacts with skin cells [12,13,14,15,16,17,18,19,20,21,22,68,69,70]. These variables can independently affect cell viability, migration, redox assays, cytokine release, A2A/salvage-related readouts, ECM markers, barrier markers, and apparent wound-repair activity.

Practical characterization should be organized as an analytical decision tree. First, DNA identity and nominal dose should be established using fluorometric DNA quantification, preferably distinguishing dsDNA and ssDNA when relevant, because UV absorbance can be inflated by RNA, phenolics, salts, or other UV-absorbing plant residuals [21,22]. Second, molecular-weight distribution and base-pair range should be profiled by agarose gel electrophoresis, capillary electrophoresis, Bioanalyzer/TapeStation analysis, or size-exclusion chromatography according to the expected fragment range and required resolution [21,22,68]. Third, DNA integrity should be tested not only in the starting material but also after sterilization, storage, nuclease challenge, and release from the delivery matrix, because the biologically exposed fraction may differ from the input PN/PDRN material [68,70]. Fourth, purity assessment should distinguish intact DNA fragments from residual RNA, DNA-bound or co-purified proteins, free nucleotides/nucleosides, degradation products, residual polysaccharides, phenolics, endotoxin/pyrogen burden, and bioburden [21,22,68,69,70].

Batch-to-batch consistency should be evaluated using repeated production lots generated through the same source-process workflow and, when possible, different harvest or culture batches. A practical comparability package should include lot-level DNA yield, dsDNA/ssDNA ratio, molecular-weight distribution, base-pair profile, A260/280 and A260/230 ratios, RNA carryover, free nucleotide/nucleoside content, residual protein, polysaccharide and phenolic burden, endotoxin/sterility status, nuclease-stability profile, and release kinetics if a carrier is used [12,13,14,15,16,17,18,19,20,21,22,23,68,69,70]. Overlaying electrophoretic or chromatographic fragment profiles across lots, together with a small bioactivity-bridging panel such as viability, migration, ECM marker expression, barrier-marker expression, or inflammatory/redox readouts, would help distinguish reproducible PN/PDRN activity from single-batch composition or contaminant effects [12,13,14,15,16,17,18,19,20,21,22,23,68,69,70]. The experimental methods according to the characterization target were summarized in Table 7.

**Table 7:** Practical characterization workflow for plant-derived PN/PDRN and delivery-controlled skin biomaterials.

Characterization Target	Practical Methods	Bioactivity or Batch-Relevance
DNA dose and identity	Fluorometric dsDNA/ssDNA assays plus UV A260 with A260/280 and A260/230 ratios [21,22].	Prevents false dose matching when UV absorbance is inflated by RNA, phenolics, salts, or free nucleotides.
Molecular-weight distribution/bp range	Agarose gel, capillary electrophoresis, Bioanalyzer/TapeStation, or size-exclusion chromatography [21,22,68].	Defines low-, medium-, and high-MW fractions that influence diffusion, release, nuclease susceptibility, tissue retention, and potency comparison.
DNA integrity after processing	Fragment analysis before/after sterilization, storage, DNase challenge, and matrix release [68,70].	Distinguishes intact-fragment effects from degradation-product effects and tests carrier-mediated protection.
Strand state	ssDNA/dsDNA assays, strand-sensitive dyes, nuclease digestion, and electrophoretic profiling [21,22].	May affect charge density, matrix binding, nuclease sensitivity, and nucleic-acid sensing risk.
RNA carryover	RNA-specific quantification, RNase treatment, and post-RNase fragment analysis [21,22,72].	Prevents misattribution of RNA-associated or double-stranded RNA-like effects to DNA-derived PN/PDRN.
Protein/histone-like nucleoprotein contamination	BCA/Bradford, SDS-PAGE, immunoblotting, or proteomics when needed [21,22].	Residual proteins may alter uptake, inflammation, cytotoxicity, and UV purity interpretation.
Free nucleotides, nucleosides, and degradation products	HPLC or LC-MS profiling of nucleotides, deoxyribonucleotides, nucleosides, and short oligonucleotides [21,22].	Important for A2A/salvage-pathway interpretation independent of intact DNA fragments.
Plant residuals	Carbohydrate assay, phenolic assay, pigment/salt/osmolality or conductivity testing [21,22,69].	Plant contaminants can confound Nrf2/redox, NF-kB/cytokine, viability, and migration assays.
Endotoxin/pyrogen/sterility	Endotoxin or pyrogen testing, sterility testing, and bioburden controls [21,22,88,89,90].	Essential for cytokine, macrophage, inflammation, and translational interpretation.
Delivery-matrix behavior and lot consistency	Rheology, swelling, degradation, release kinetics, released-fraction DNA profiling, fragment-profile overlay, and bioactivity-bridging assays across lots [23,24,25,26,27,28,29,30,31,32,33,34,35,36,37,38,39,40,41,42,43,44,45,46,47,48,49,50,51,52,53,54,55,56,57,58,59,60,61,62,63,64,65,66,67,68].	Distinguishes free-DNA activity from carrier-mediated exposure and separates reproducible material behavior from single-batch artifacts.

## 8. Skin Cell and Tissue Endpoints for Delivery-Controlled Plant PN/PDRN

Studies on skin aging should distinguish between collagen-restoring activity and platform-induced hydration or optical smoothing. Fine-wrinkle hypotheses related to fine wrinkles are most appropriately supported by dermal fibroblast assays for COL1A1, COL3A1, elastin, fibronectin, MMP/TIMP balance, and photoaging markers [9,15,23,79,80]. When possible, these assays should be followed by 3D skin or ex vivo validation to connect cellular markers with tissue-level repair [91,92,93,94,95,96,97].

Clinical evidence for PN/PDRN remains dominated by animal-derived products and aesthetic PN/PDRN studies, whereas plant-derived PN/PDRN skin data are largely preclinical [3,4,5,6,7,8,9,10,11]. Wound repair should be discussed primarily as an animal-derived PDRN benchmark and general wound-biology indication [3,4,5,6,7,8,91,92,93,94,95,96,97,98,99]. Skin rejuvenation and pigmentation should be framed using aesthetic PN/PDRN and pigmentation/photoaging studies [9,10,11,79,80,81,82]. Scar modulation, barrier repair, and sensitive skin hypotheses should be treated as extension areas supported mainly by fibrosis, wound, and skin barrier biology unless direct plant-derived clinical data become available [83,84,85,100,101,102,103,104,105,106].

Barrier and sensitive skin hypotheses should involve keratinocyte differentiation and barrier endpoints such as FLG, IVL, loricrin (LOR), transglutaminase 1 (TGM1), CLDN1, occludin (OCLN), zonula occludens-1 (ZO-1), AQP3, and transepithelial electrical resistance (TEER). Plant-derived PN/PDRN studies support the relevance of FLG, IVL, CLDN1, and AQP3 readouts, whereas general skin barrier literature supports cornified-envelope, tight-junction, and hydration markers [12,13,14,100,101,102,103,104,105,106].

Wound-healing-related studies should incorporate migration, re-epithelialization, angiogenic support, inflammatory resolution, and matrix remodeling. Classical wound biology emphasizes keratinocyte migration, fibroblast proliferation, collagen remodeling, angiogenesis, and inflammatory transition as coordinated events, rather than isolated collagen production [91,92,93,94,95,96,97,98,99].

Hypertrophic scars or scar modulation hypotheses require additional caution because regenerative ECM deposition and fibrotic overproduction are biologically different. Scar-focused studies should include TGF-β/Smad, α-SMA/ACTA2, CTGF, LOX/LOXL2, COL1/COL3 ratio, MMP/TIMP balance, inflammatory markers, and collagen gel contraction or scar fibroblast models [83,84,85].

## 9. Future Perspectives and Conclusions

Future studies should move from feasibility reporting toward standardized, hypothesis-driven comparisons. A minimum standardization package should include source identity, culture or harvest conditions, extraction and fragmentation method, purification and sterilization process, DNA mass, molecular weight distribution, base-pair range, strand state, RNA carryover, free nucleotide/nucleoside fraction, residual protein or histone-like nucleoprotein content, polysaccharide and phenolic burden, endotoxin/pyrogen and sterility status, nuclease stability, release kinetics, and stability after storage or sterilization [12,13,14,15,16,17,18,19,20,21,22,23,68,69,70]. Comparative studies with animal-derived PDRN should be designed only after these variables are matched or explicitly reported, using equivalent DNA dose, fragment-size distribution, purity, degradation profile, delivery platform, and exposure duration [1,2,3,4,5,6,7,8,12,13,14,15,16,23,33,34,49,50,55,69].

Regulatory-development planning should also be introduced before findings are extrapolated toward translational use. The appropriate evaluation pathway will depend on intended use, route of administration, depth and duration of tissue contact, and whether the product is positioned as a topical cosmetic ingredient, a skin-contact biomaterial, a wound dressing, a microneedle-based delivery system, an injectable matrix, or a drug/device-like combination product [88,89,90,107,108]. For skin-contact or device-like platforms, biological safety evaluation should be aligned with contact type and exposure duration using risk-based biocompatibility principles such as ISO 10993-1 and relevant regulatory guidance; reconstructed human epidermis irritation testing, sensitization-relevant assays, cytotoxicity testing, sterility/endotoxin testing, release/stability studies, and product-specific efficacy endpoints should be selected according to the intended route and proposed use [88,89,90,107,108].

Clinically relevant evaluation models should progress beyond monoculture screening. A staged model ladder may include keratinocyte and fibroblast assays, photoaged human dermal fibroblasts, 3D reconstructed skin, TEER/permeability barrier models, ex vivo human skin or wound models, endothelial/macrophage co-culture systems, and disease-relevant diabetic-wound or scar models [79,80,83,84,85,86,87,91,92,93,94,95,96,97,98,99,100,101,102,103,104,105,106]. Early human studies, when justified, should use route- and indication-specific endpoints such as tolerability, adverse events, trans-epidermal water loss, hydration, elasticity, validated wrinkle or scar scales, wound closure kinetics, imaging, histology, and biomarker panels rather than relying solely on short-term cell-proliferation or collagen-expression assays.

The current evidence bases for plant-derived PN/PDRN remain preclinical. Plant-source DNA fractions may become useful skin biomaterials only if molecular identity, compositional purity, residual-contaminant burden, delivery behavior, pathway dependence, and reproducibility are defined with sufficient rigor. Delivery platforms such as HA matrices, alginate or oxidized alginate hydrogels, chitosan complexes, nanocellulose scaffolds, and microneedles remain promising, but their effects must be separated from DNA-specific effects through free-DNA, carrier-only, loaded-platform, DNase/RNase-treated, process-blank, and matched-comparator arms.

In conclusion, plant-derived PN/PDRN should not yet be considered clinically or mechanistically equivalent to animal-derived PDRN. The available literature supports feasibility and partially overlapping biological signatures, but direct comparative and perturbation-based evidence remains limited. In particular, A2A receptor-related signaling should be regarded as a candidate working hypothesis for plant-derived PN/PDRN unless receptor dependency is demonstrated using antagonist, knockdown, DNA-degradation, and matched-comparator experiments. Plant-derived PN/PDRN is therefore best interpreted as a delivery-dependent DNA biomaterial class whose biological meaning depends on source control, molecular characterization, compositional purity, platform testing, reproducibility, and hypothesis-driven biological validation.

## Data Availability

No new data were created or analyzed in this study. Data sharing is not applicable to this article.
